
JOURNAL INFORMATION
==============================
NLM Title Abbreviation: ScientificWorldJournal
Journal ID: ScientificWorldJournal
NLM Journal ID: 101131163
Journal ID: TSWJ

The Scientific World Journal

ISSN: 2356-6140
EISSN: 1537-744X
Publisher: Wiley

ARTICLE INFORMATION
==============================
PMCID: PMC6083989
PMID: 30147624
DOI: 10.1100/tsw.2001.244
Article ID: 356234
Article version: 1
Subjects: Short Report

MRG 15, A Chromodomain Protein Related to Mortality Factor 4 (MORF 4) is Involved in Multiple Complexes

Pardo Patricia S. 1 *
Bick Roger J. 2
Smith Olivia Pereira 1
1 Huffington Center on Aging, Baylor College of Medicine, One Baylor Plaza N820, USA
2 Department of Pathology, The University of Texas Medical School, Houston, TX 77030, USA
*Corresponding author.
Publication date: 2001
Electronic publication date: 2001 Nov 2
Volume: 1
First page: 147
Last page: 147
Copyright: Copyright © 2001 Patricia S. Pardo et al.
Copyright year: 2001
License: This is an open access article distributed under the Creative Commons Attribution License, which permits unrestricted use, distribution, and reproduction in any medium, provided the original work is properly cited.
License URL: https://creativecommons.org/licenses/by/3.0/

Funding: Doris and Curtis Hankamer Foundation NIA R37AG05333

==============================
Miami *Nature Biotechnology* Short Reports

*TheScientificWorld* (2001) 1 (S3), 147SR

ISSN 1532-2246; DOI 10.1100/tsw.2001.244

### MRG 15, A CHROMODOMAIN PROTEIN RELATED TO MORTALITY FACTOR 4 (MORF 4) IS INVOLVED IN MULTIPLE COMPLEXES

Patricia S. Pardo*[1], Roger J. Bick[2], and Olivia Pereira Smith[1]

[1]Huffington Center on Aging, Baylor College of Medicine, One Baylor Plaza N820 and
[2]Department of Pathology, The University of Texas Medical School, Houston, TX 77030, USA
* Corresponding author.

**INTRODUCTION.** Replicative senescence, the final non-proliferative state reached by normal cells when cultured in vitro, is considered a model of aging process at the cellular level. Senescence is dominant over indefinite division (1) and analysis of hybrids obtained by fusion of immortal human cell lines has defined four complementation groups (A-D) for indefinite division (2). Genes responsible for senescence are located on human chromosomes 1, 4 and 7 for complementation groups C, B and D, respectively. The mortality factor 4 (MORF 4) gene, cloned in our laboratory, is on chromosome 4 and is capable of inducing senescence in cell lines from group B with no effect on cell lines from the other groups (3). Two MORF 4 related genes, MRG X and MRG 15, have been cloned. These genes are of interest as they are induced by serum stimulation. The three proteins have common features, nuclear localization signal, helix-loop-helix and leucine zipper motifs. MRG 15 is of particular interest, as it has an N-terminal extension harboring a chromodomain. Using the yeast two-hybrid system a novel protein, PAM (protein associated with MRG), has been identified as an interacting partner of MRG 15 (Leung and Pereira Smith, unpublished results). We have determined that there are at least two different nucleoprotein complexes that involve MRG 15 and identified some of the interacting partners.

**METHODS.** Stable clones expressing HA-tagged versions of MRG 15 and PAM following transfection of EJ cells were isolated for these studies. Fractionation of nucleoprotein complexes was done by sedimentation on 5 20% sucrose gradients. Fractions were analyzed by Western blot using an anti-HA antibody (Santa Cruz Biotechnology, Inc.) or an antibody raised against a peptide from the N-terminal extension of MRG 15. The same nuclear extracts were analyzed by IP-Western to assess the interactions.

GFP-tagged versions of native MRG 15 and deletion mutants lacking the chromodomain or the leucine zipper region were transfected into the stable-expressing PAM-HA clone using Lipofectamine plus (GIBCO BRL products) according to manufacturer instructions. Nucleoprotein complexes containing the native and mutant MRG 15 proteins were analyzed by sucrose gradients, and colocalization determined by confocal microscopy.

**RESULTS.** Analysis of fractions from sucrose gradients of nuclear extracts from cells stably-expressing MRG 15-HA have detected two protein complexes of 3.5 and 14 S. PAM is associated with the 3.5 S complex. The studies with deletion mutants indicate that the leucine zipper region is involved in the interaction with PAM. Confocal microscopy of cells expressing PAM-HA that had been transfected with MRG 15-GFP has demonstrated that PAM-HA colocalizes only partially with MRG 15, confirming the latter is part of more than one multiprotein complex. Characterization of other proteins of the complexes is in progress.

**ACKNOWLEDGEMENTS.** This work was supported by the Doris and Curtis Hankamer Foundation and a grant from NIA R37AG05333.

**REFERENCES.**

1. Pereira Smith, O.M. and Smith, J.R. (1983) Science 221, 964-966
2. Pereira Smith, O.M. and Smith, J.R. (1988) Proc. Natl. Acad. Sci. U S A 85, 6042-6046
3. Bertram, M.J., Berube, N.G., Hang-Swanson, X., Ran, Q., Leung, J.K., Bryce, S., Spurgers, K., Bick, R.J., Ning, Y., Clark, L.J., Parkinson, E.K., Barrett, J.C., Smith, J.R., and Pereira Smith, O.M. (1999) Mol. Cell. Biol. 19, 1479-1485
